# Research Progress on Targeted Antioxidant Therapy and Vitiligo

**DOI:** 10.1155/2022/1821780

**Published:** 2022-03-14

**Authors:** Jingzhan Zhang, Wen Hu, Peng Wang, Yuan Ding, Hongjuan Wang, Xiaojing Kang

**Affiliations:** ^1^Department of Dermatology, People's Hospital of Xinjiang Uygur Autonomous Region, Urumqi, 83001, China; ^2^Xinjiang Clinical Research Center for Dermatologic Diseases, Urumqi, 83001, China; ^3^Xinjiang Key Laboratory of Dermatology Research, Urumqi, 83001, China; ^4^Graduate School of Xinjiang Medical University, Urumqi, 83001, China; ^5^College of Life Science and Technology, Xinjiang University, Urumqi, 83001, China

## Abstract

Vitiligo is a common acquired depigmenting disease characterized by the loss of functional melanocytes and epidermal melanin. Vitiligo has a long treatment cycle and slow results, which is one of the most difficult challenges for skin diseases. Oxidative stress plays an important role as an initiating and driving factor in the pathogenesis of vitiligo. Antioxidant therapy has recently become a research hotspot in vitiligo treatment. A series of antioxidants has been discovered and applied to the treatment of vitiligo, which has returned satisfactory results. This article briefly reviews the relationship between oxidative stress and vitiligo. We also describe the progress of targeted antioxidant therapy in vitiligo, with the aim of providing a reference for new drug development and treatment options for this condition.

## 1. Introduction

Vitiligo is the most common skin depigmentation disease and is characterized by depigmentation of the skin or mucous membranes. Its incidence rate is 0.5%–2.0% [1], and it affects the appearance and readily causes mental illnesses such as anxiety and depression in patients [2]. Clinically, it is often accompanied by various autoimmune diseases such as hyperthyroidism, diabetes, and alopecia areata; moreover, the disease is persistent and readily relapses, and treatment is difficult [3]. The pathogenesis of vitiligo is complex and involves oxidative stress, immune, genetic, and neuropsychiatric factors [4, 5]. Targeted drugs for immune-related pathways have been actively developed, such as the phosphodiesterase-4 inhibitor apremilast and the JAK inhibitors ruxolitinib and tofacitinib, which have all been used in the targeted treatment of vitiligo [6–8].

Oxidative stress is the initial pathogenic trigger factor of melanocyte degeneration in vitiligo patients and plays a crucial role in disease occurrence and development [9–11]. Reducing the level of oxidative stress in patients with vitiligo is an effective method for its treatment. Some traditional antioxidants for the treatment of vitiligo include pseudocatalase, vitamin E, coenzyme Q *α*-lipoic acid, and zinc [10, 12, 13]. Although these antioxidants have certain curative effects, their effect is limited, targeting is not robust, and many treatment methods lack evidence-based data. In recent years, with the in-depth study of oxidative stress in the pathogenesis of vitiligo, some key molecules and regulatory signaling pathways related to oxidative stress have been found. More researchers are committed to research on antioxidative stress-targeting drugs that affect the skin. This report reviews the antioxidants and their antioxidation-related signaling pathways that are currently exploited clinically or potentially under study, to provide a new direction for the treatment of vitiligo.

## 2. Oxidative Stress and Vitiligo

Oxidative stress refers to the excessive production of highly active molecules such as reactive oxygen species (ROS) in the body when the body is stimulated by various harmful factors and when the degree of oxidation exceeds the scavenging of oxides. Here, the oxidation system and antioxidant system are unbalanced, resulting in tissue damage. ROS includes O^2−^, -OH, and H_2_O_2_. They are the main index used to evaluate the level of oxidative stress [14, 15]. The antioxidant defense system plays an important role in protecting cells from oxidative stress and damage. Antioxidants such as glutathione, ascorbic acid, and thioredoxin and antioxidant enzymes such as superoxide dismutase (SOD), glutathione-S-transferase (GST), glutathione peroxidase (GPx), and catalase (Cat) control oxidative stress and protect proteins, lipids, and DNA [16–18]. In a normal physiological state, the small amount of ROS produced by the body is converted into nontoxic substances by the antioxidant system, such that it will not cause harm to the human body. However, ROS production can increase under pathological conditions, such as inflammation, cancer, and exposure to exogenous factors, such as ultraviolet rays or chemicals, causing tissue and cell damage.

**Figure 1:**
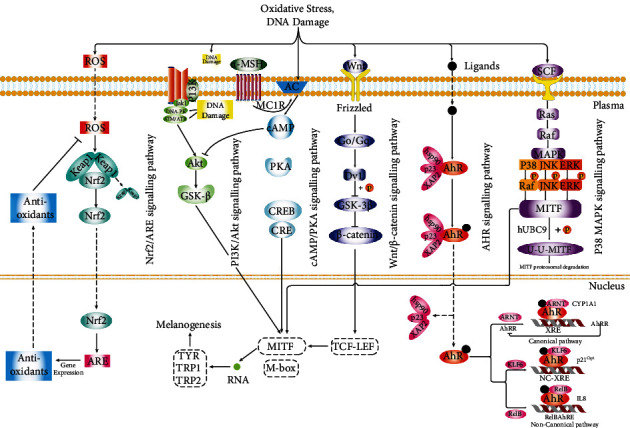
The signaling pathways involved in vitiligo oxidative stress. ROS: reactive oxygen species; Nrf2: nuclear factor-E2-related factor 2; ARE: antioxidant response element; PI3K: phosphoinositide 3-kinase; Akt: protein kinase B; GSK-3*β*: glycogen synthase kinase 3*β*; MC1R: melanocortin 1 receptor; AC: adenylyl cyclase; CREB: cAMP-response element binding protein; CRE: cAMP-response element; MITF: microphthalmia-associated transcription factor; TYR: tyrosinase; TRP-1: tyrosinase-related protein 1; TRP-2: tyrosinase-related protein 2; Wnt: wingless/integrated; Go/Gq: G protein; Dvl: Dishevelled; TCF-LEF: T cell factor-lymphoid enhancer factor; AhR: aromatic hydrocarbon receptor; AhRR: AhR repressor; Hsp90: heat shock protein 90; p23: co-chaperone protein; XAP-2: HBV X-associated protein 2; SCF: stem cell factor; MAPK: mitogen-activated protein kinase; ARNT: aryl hydrocarbon receptor nuclear translocator; XRE: xenobiotic-responsive element; KLF6: Kruppel-like factor 6; JNK: c-Jun N-terminal kinase; ERK: extracellular signal-regulated kinase; CYP1A1: cytochrome P450 1A1.

The skin is the largest organ in contact with the environment. Melanocytes are likely to produce excessive ROS when melanin is synthesized after ultraviolet irradiation or inflammation [19]. ROS can attack melanocytes, thereby interfering with their normal proliferation, metabolism, and differentiation, inducing immune responses and causing apoptosis, leading to localized or generalized skin depigmentation [20]. In addition, oxidative stress can also increase the synthesis of intermediate toxic products from melanin, thus promoting catecholamine release and causing immune damage to melanocytes [21]. The ROS and malondialdehyde (MDA) levels in the blood and skin lesions of vitiligo patients are significantly increased. MDA can increase ROS levels by enhancing the activity of mitochondrial enzymes and the respiratory chain complex. High ROS levels can increase the levels of cytokines, such as interleukin-2 (IL-2) and B cell lymphoma-2 (Bcl-2), which can upregulate the expression of antiapoptotic proteins, convert T cells to an antiapoptotic phenotype, and aggravate melanocyte oxidative injury [22–24]. With the in-depth study of the pathogenesis of vitiligo, it was found that this is related to antioxidant signaling pathways, including nuclear factor-E2-related factor 2 (Nrf2)/antioxidant response element (ARE), phosphatidylinositol-3-kinase-Akt (PI3K-Akt), wingless/integrated (Wnt)/*β*-catenin, aromatic hydrocarbon receptor (AhR), and p38 mitogen-activated protein kinases (p38 MAPKs) (Figure 1). An increasing number of new targeted antioxidants have been found, which provides broader approaches for treating vitiligo with antioxidants (Table 1).

### 2.1. Antioxidant Therapy Targeting the Nrf2/ARE Pathway

The Nrf2/ARE signaling pathway is an important antioxidant pathway identified in recent studies. It plays an important role in protecting melanocytes from antioxidant stress [25]. Nrf2 belongs to the CNC family of basic leucine zipper (bZIP) transcription factors and is responsible for protecting cells from oxidative damage [26]. The bZIP structure of Nrf2 can bind to AREs and initiate the expression of downstream antioxidant proteins [27]. The ARE is a specific DNA promoter-binding sequence. It can be activated by a variety of oxidative substances, regulate the expression of downstream SOD and other protective genes, and play a role in maintaining the normal functions of the body [28]. Under excessive oxidative stress, melanocytes exhibit reduced Nrf2 nuclear translocation and transcriptional activity, resulting in decreased expression of antioxidant enzymes, such as heme oxygenase-1 (HO-1), which results in melanocyte apoptosis [29, 30]. Dysfunction of the Nrf2 signaling pathway can lead to increased sensitivity of vitiligo melanocytes to H_2_O_2_-induced oxidative damage [31]. The Nrf2/ARE axis can also regulate the expression of anti-inflammatory genes and inhibit the progression of inflammation [32]. Many antioxidant drugs targeting the Nrf2/ARE pathway have been identified, such as simvastatin, aspirin, *Ginkgo biloba* extract (EGb761), berberine, baicalein, ginsenoside Rk1, cinnamaldehyde, and Nle4-D-Phe7-*α*-MSH (afamelanotide). EGb761, berberine, baicalein, ginsenoside Rk1, and cinnamaldehyde are natural antioxidants found in plants.

EGb761 has significant therapeutic effects on various oxidative stress-related diseases, such as Alzheimer's disease, stroke, and cardiovascular disease [33]. Further, EGb761 can protect melanocytes from oxidative stress by activating antioxidant enzymes and inhibiting endoplasmic reticulum stress [34]. Parsad et al. found that EGb761 has a clear effect on localized vitiligo. In this study, the treatment group was administered EGb761 43 times daily for 6 months. The expansion of leukoplakia in the treatment group was significantly slower or halted compared to that in the placebo group [35]. Zhang et al. found that the antioxidant effect of EGb761 *in vitro* was achieved by activating Nrf2 and its downstream antioxidant genes. After inhibiting the Nrf2 signaling pathway, the protective effect of EGb761 on oxidative damage of melanocytes was reduced [36]. Jiang et al. pretreated human melanocytes with berberine, showing that it activates the Nrf2 signaling pathway, promotes the expression of the downstream antioxidant genes *HO-1* and *SOD*, enhances the function of melanocytes to synthesize melanin, and improves the activation of nuclear factor- (NF-) *κ*B to protect human melanocytes from H_2_O_2_-induced oxidative stress [37]. Uchi et al. found that cinnamaldehyde can activate Nrf2 and induce its nuclear translocation, resulting in the upregulation of *HO-1* gene expression [38]. Cinnamaldehyde also activates the Nrf2/HO-1 antioxidant system and was found to alleviate benzopyrene-induced ROS production in keratinocytes [39, 40]. More than a hundred Kampo formulations, including cinnamaldehyde, have been approved by the Japanese Ministry of Health as prescription drugs for the treatment of various chronic diseases; cinnamaldehyde, in particular, may have the potential to treat vitiligo and other diseases caused by oxidative stress [38, 41]. Baicalein can enhance the cellular antioxidant defense ability of melanocytes in patients with vitiligo by activating the Nrf2 signaling pathway [42]. The latest research further found that ginsenoside Rk1 can protect melanocytes from oxidative stress induced by H_2_O_2_ by regulating the expression of the Nrf2/HO-1 protein [43].

Simvastatin is a hydroxymethylglutaryl CoA reductase inhibitor with antioxidant capacity. Simvastatin protects human melanocytes from H_2_O_2_-induced oxidative stress by activating Nrf2, whereas downregulating Nrf2 mitigates the protective effect of simvastatin on H_2_O_2_-induced oxidative damage, which supports the contention that simvastatin is a potential therapeutic drug for vitiligo [44, 45]. At present, *in vitro* and *in vivo* animal studies have shown good antioxidant therapeutic effects, but clinical studies have failed to exhibit similar significant improvement in skin lesions of vitiligo patients with oral administration of simvastatin. This may be because higher doses are required for its effectiveness. It is anticipated that the topical therapy can provide a sufficiently high local concentration, for which a topical simvastatin salt trial (EVRAAS trial) is underway [46, 47]. Aspirin has antioxidant activity and has good preventive and therapeutic effects against many oxidative stress-related diseases. Zailaie randomly divided 32 patients with nonsegmental progressive vitiligo into two groups. One group took 300 mg/day aspirin, whereas the other group took a placebo. After 12 weeks, the peripheral blood monocytes of the patients in both groups were stimulated by lipopolysaccharide. With this, IL-1 *β*, IL-6, IL-8, anti-melanocyte antibody, tumor necrosis factor- (TNF-) *α*, and soluble IL-2 receptor decreased significantly. All patients in the aspirin treatment group showed reduced vitiligo progression, and two patients completely recovered [48]. Jian et al. found that aspirin significantly induces Nrf2 nuclear translocation, increases pNrf2 and total Nrf2 levels, and induces HO-1 expression in human melanocytes. In addition, the inhibition of Nrf2 or HO-1 expression alleviates the protective effect of aspirin on melanocytes and other protective effects on H_2_O_2_-induced cytotoxicity and apoptosis [49]. These results suggest that aspirin protects human melanocytes from H_2_O_2_-induced oxidative stress through Nrf2-driven transcriptional activation of HO-1. Subcutaneous injection of the alpha-melanocyte stimulating hormone analogue afamelanotide can increase the level of Nrf2 in melanocytes and keratinocytes, thereby reducing ROS levels and local inflammation [50]. Dimethyl fumarate can also play an antioxidant role through the Nrf2 pathway. The current clinical research has mainly focused on the treatment of multiple sclerosis [51]. However, it might also have certain therapeutic significance for vitiligo, and further research is needed [52].

### 2.2. Antioxidative Therapy Targeting the PI3K/Akt Pathway

The PI3K/Akt signaling pathway plays an important role in cell proliferation, differentiation, metabolism, and apoptosis. After activation of the PI3K/Akt signaling pathway, apoptosis can be inhibited via several mechanisms [53–57]. The PI3K/Akt signaling pathway can regulate the expression of antioxidant enzymes, such as SOD, Cat, GPX, and HO-1 [30, 58]. Activation of the PI3K/Akt signaling pathway can increase the expression of Bcl-2 protein and inhibit melanocyte apoptosis induced by oxidative stress [4, 19]. The Bcl-2 and caspase protein families are downstream of the PI3K/Akt signaling pathway and are key proteins involved in oxidative stress-induced apoptosis [20]. Antioxidants such as quercetin, geniposide, 8-methoxypsoralen, and chalcones can inhibit melanocyte apoptosis induced by oxidative stress by regulating PI3K/Akt.

Quercetin, a flavonol glycoside, has a strong antioxidant effect. Yang et al. showed that quercetin protects human melanocytes from H_2_O_2_-induced apoptosis by regulating PI3K/Akt and p38 signaling [59]. The effects of geniposide on cell viability, apoptosis, and antioxidant enzyme activity can be inhibited by the PI3K inhibitor LY294002, indicating that geniposide has an antioxidant effect by activating PI3K/Akt signaling. Geniposide can reduce ROS accumulation and prevent apoptosis induced by oxidative stress by promoting the activity of several antioxidant enzymes, such as HO-1, Cat, and SOD [60, 61]. 8-Methoxypsoralen is a furanocoumarin, which has been widely used in the treatment of vitiligo or hyperproliferative skin disorders, such as psoriasis. It can reduce AKT phosphorylation, scavenge oxygen free radicals, and decrease apoptosis [62]. Flavonoid compounds such as chalcones extracted from Kaliziri can activate PI3K/Akt and GSK3*β* signaling pathways, increase tyrosinase (TYR) activity, promote the formation of epidermal melanin, and induce the recoloration of vitiligo [63]. Mesenchymal stem cells (MSCs) can target the PTEN/PI3K/Akt pathway to regulate melanocyte proliferation and apoptosis. MSCs could thus be a promising method for the treatment of vitiligo [64]. Some studies have found that basic fibroblast growth factor promotes melanocyte migration and cytoskeletal rearrangements through PI3K/Akt and ERK signaling pathways, which might have certain clinical application value in melanocyte transplantation [65, 66].

### 2.3. Antioxidative Therapy Targeting the Wnt/*β*-Catenin Pathway

The Wnt/*β*-catenin signaling pathway activates the expression of target genes in the nucleus and controls cell proliferation, differentiation, and apoptosis [67]. Wnt signaling plays a key role in the differentiation of melanocyte stem cells, and Wnt1 and Wnt3a promote the differentiation of neural crest stem cells into premelanocytes [68–70]. Mei et al. found that the *Wnt5a* gene in the canonical Wnt/*β*-catenin pathway can promote melanocyte differentiation and proliferation [71]. Further, the Wnt/*β*-catenin pathway regulates the expression of cadherin in epithelial cells. Decreased cadherin expression in epithelial cells of patients with vitiligo results in a decrease in adhesion of these cells to the basement membrane during oxidative stress [72]. In addition, the Wnt/*β*-catenin signaling pathway might be involved in the activation of microphthalmia-associated transcription factor (MITF) and melanin synthase in vitiligo [73]. Regazzetti et al. found that in vitiligo lesions, oxidative stress decreases the expression and activation of Wnt in melanocytes. Wnt/*β*-catenin signaling inhibits H_2_O_2_-induced oxidative damage in keratinocytes and melanocytes, whereas Wnt agonists can trigger melanocyte differentiation and melanogenesis in vitiligo [74]. These findings show that stimulating the Wnt signaling pathway could be an adjuvant treatment for vitiligo.

Secreted frizzled-related protein 5 (SFRP5) is a member of the highly conserved secreted curl-related protein family [75] and is very similar to the Frizzled (Fz) receptor in the Wnt signaling pathway, and thus, it can inhibit Wnt signaling via competitive inhibition with the Fz receptor [76]. Based on this, SFRP5 was identified as an inhibitor of the Wnt signaling pathway [77, 78]. Glycogen synthase kinase-3*β* (GSK-3*β*) is a negative regulator of the Wnt/*β*-catenin signaling pathway. SKL2001 is an agonist of the Wnt/*β*-catenin signaling pathway. The transcription of *β*-catenin can be upregulated by increasing the level of intracellular *β*-catenin protein. Some scholars found that inhibiting GSK-3*β* or using a Wnt/*β*-catenin inducer (SKL2001) can activate the Wnt/*β*-catenin pathway [79–81]. The antioxidant properties of vitamin D have also been investigated in various human cells, such as cardiomyocytes, endothelial cells, gastric epithelial cells, and melanocytes. Previous studies found that MDA levels in vitiligo patients are elevated, whereas vitamin D levels are low. Interestingly, the serum vitamin D levels in vitiligo patients are negatively correlated with serum MDA levels, suggesting that vitamin D has a potential antioxidant effect [82]. Recent studies found that H_2_O_2_-induced oxidative damage and inhibition of the Wnt/*β*-catenin signaling pathway can be reversed by vitamin D [83]. Therefore, vitamin D can activate the Wnt/*β*-catenin signaling pathway to protect human melanocytes from oxidative damage. H_2_ has strong antioxidant activity and can reverse the melanocyte apoptosis and dysfunction induced by H_2_O_2_. H_2_ positively regulates *β*-catenin in melanocytes treated with H_2_O_2_, and the *β*-catenin pathway is related to H_2_-induced Nrf2 activation. H_2_ might thus be a promising therapeutic agent for the antioxidant treatment of vitiligo [84]. Adipose stem cell transplantation could also be an innovative regenerative method used for the treatment of vitiligo. The adipose tissue extracellular fraction can promote activation of the Wnt/*β*-catenin pathway and improve the ability of melanocytes to resist oxidative stress through intracellular antioxidant enzymes [85].

### 2.4. Antioxidant Therapy Targeting the Aromatic Hydrocarbon Receptor (AhR) Pathway

AhR is a ligand-activated transcription factor, is involved in repairing mitochondrial oxidative damage, and plays an important regulatory role in mitochondrial oxidative damage-mediated apoptosis. AhR can upregulate expression of the mitochondrial biosynthesis-related molecule Nrf1 and its downstream molecules mitochondrial transcription factor A and cytochrome C. Improper activation of the AhR signaling pathway can aggravate oxidative damage to mitochondria and melanocytes, whereas regulating the AhR signaling pathway can increase the number of mitochondria and restore mitochondrial function [86].

Luecke et al. first confirmed the existence of a functional AhR signaling pathway in human melanocytes [87]. Schallreuter et al. found that the endogenous ligand of AhR is decreased significantly in the skin lesions of vitiligo patients. Further, this group found that the expression of AhR and its downstream target genes encoding cytochrome P450 1A1 and cyclooxygenase 2 is decreased in the skin lesions of vitiligo patients [88]. Other studies showed that the expression levels of antioxidant molecules downstream of AhR, such as HO-1, GST, GPx, CAT, and SOD, are also significantly reduced in vitiligo [29]. The expression of AhR in peripheral blood mononuclear cells of vitiligo patients is decreased and is closely related to disease severity [89]. AhR agonists are the active ingredients in some traditional herb formulations for vitiligo [90]; to illustrate, tapinarof, isopsoralen, and norisoboldine are potentially new targets for its treatment [91–93]. In addition to activating the PI3K/Akt pathway, cinnamaldehyde might inhibit abnormal activation of the AhR signaling pathway, reduce the production of ROS in keratinocytes, and could have a therapeutic effect on vitiligo [38].

### 2.5. Antioxidant Therapy Targeting the p38 MAPK Pathway

The p38 MAPK signaling pathway is an important signal transduction pathway that can respond to oxidative stress. The p38 MAPK pathway can increase the expression of ommatidium-related transcription factor (MITF) to upregulate melanin production [94, 95]. Hyperacetylated epigallocatechin gallate (EGCG) can effectively inhibit p38 MAPK phosphorylation induced by H_2_O_2_, significantly reducing ROS production, restoring disruptions to the mitochondrial membrane potential, and reducing melanocyte apoptosis [96]. In a monophenone-induced vitiligo animal model, EGCG can delay depigmentation time and reduce the incidence and area of depigmentation [97]. Additionally, a clinical study confirms that topical EGCG is effective in treating vitiligo patients [98]. 2′,3,4,4′-Tetrahydrochalcone (RY3-a), which is isolated from wild-type cotton bollworm seeds, has good melanogenesis and antioxidant activity. Its analogue, RY3-c, has better melanogenesis and antioxidant activity and lower toxicity. Mechanistic studies have shown that RY3-c can repair cell damage caused by excessive oxidative stress by activating the MAPK pathway [99]. Further, flumequine can induce an increase in the melanin content of zebrafish larvae and B16F10 cells by activating p38 MAPK and c-Jun N-terminal kinase (JNK), and this has the potential for use as an antivitiligo drug [100]. Maclurin can activate the p38 MAPK/CREB and cAMP/PKA/CREB signaling pathways and increase the expression of MITF genes to have an antioxidant effect on promoting melanogenesis [101]. Minocycline significantly inhibits the activation of JNK, p38 MAPK, and caspase 3 induced by H_2_O_2_ and can be used to prevent the loss of melanocytes in the early stage of vitiligo [102]. Other p38 MAPK agonists, including psoralen derivative-MPFC, baicalein, cynarine, Kursi Karwiya or caraway tablet, 1,5-dicaffeoylquinic acid, glutathione, apigenin, and methyl 3,5-di-caffeoylquinate, could also be used to treat vitiligo, as new target compounds, which needs to be further studied [103–110].

## 3. Conclusion and Prospects

Oxidative stress is involved in the occurrence and development of vitiligo. A series of antioxidants has been discovered, some of which have been used clinically, but most of the antioxidants are still limited to *in vitro* experiments, and further animal experiments and standardized clinical treatments are needed to verify their efficacy. With in-depth research on targeted antioxidant therapy, we believe that this will provide a breakthrough for vitiligo treatment.

## Figures and Tables

**Table 1:** Antioxidants and their targeted signaling pathways related to vitiligo. Potential antivitiligo.

Target pathway	Treatment goal	Clinically applied antioxidants	Experimental antioxidants	Mechanism	References
Nrf2/ARE	Reduce oxidative damage of melanocytes	Simvastatin, aspirin, *Ginkgo biloba* extract (EGb761), afamelanotide	Berberine, cinnamaldehyde, baicalein, ginsenoside Rk1, dimethyl fumarate	Upregulation of antioxidant gene expression	[33–52]
PI3K/AKT	Reduce oxidative damage of melanocytes	8-Methoxypsoralen, chalcones, mesenchymal stem cells, basic fibroblast growth factor	Quercetin, geniposide	Regulate melanocyte proliferation, differentiation, and metabolism	[59–66]
Wnt/*β*-catenin	Stimulation of repigmentation	Vitamin D	Wnt receptor inducer (SKL2001), H_2_, adipose tissue extracellular fraction (AT-Ex)	Stimulation of melanocyte stem cell proliferation, differentiation, and migration	[79–85]
AhR	Reduce oxidative damage of melanocytes	—	Tapinarof, isopsoralen, norisoboldine, cinnamaldehyde	Repairing mitochondrial oxidative damage by regulating mitochondrial biosynthesis	[38, 91–93]
p38 MAPK	Reduce oxidative damage of melanocytes	Minocycline, Kursi Karwiya or caraway tablet, 1,5-dicaffeoylquinic acid, glutathione	Hyperacetylated epigallocatechin gallate (EGCG), 2′,3,4,4′-tetrahydrochalcone (RY3-a), flumequine, maclurin, psoralen derivative-MPFC, baicalein, cynarine, apigenin, methyl 3,5-di-caffeoylquinate	Melanogenesis and antioxidant activity	[96–110]
